# Levodopa Effects on Hand and Speech Movements in Patients with Parkinson’s Disease: A fMRI Study

**DOI:** 10.1371/journal.pone.0046541

**Published:** 2012-10-09

**Authors:** Audrey Maillet, Alexandre Krainik, Bettina Debû, Irène Troprès, Christelle Lagrange, Stéphane Thobois, Pierre Pollak, Serge Pinto

**Affiliations:** 1 Université Joseph Fourier - Grenoble I, Grenoble, France; 2 Grenoble Institut des Neurosciences, INSERM-UJF-CEA-CHU U836, Grenoble, France; 3 Centre Hospitalier Universitaire (CHU), Neuroradiologie & IRM, Grenoble, France; 4 Centre Hospitalier Universitaire (CHU), Neurologie, Grenoble, France; 5 Hospices Civils de Lyon, Hôpital Neurologique, Lyon, France; 6 Centre de Neuroscience Cognitive, UMR 5229 CNRS/Université Lyon I, Lyon, France; 7 Hôpitaux Universitaires, Genève, Switzerland; 8 Laboratoire Parole et Langage, UMR 7309 CNRS/Aix-Marseille Université, Aix-en-Provence, France; Duke University, United States of America

## Abstract

Levodopa (L-dopa) effects on the cardinal and axial symptoms of Parkinson’s disease (PD) differ greatly, leading to therapeutic challenges for managing the disabilities in this patient’s population. In this context, we studied the cerebral networks associated with the production of a unilateral hand movement, speech production, and a task combining both tasks in 12 individuals with PD, both *off* and *on* levodopa (L-dopa). Unilateral hand movements in the *off* medication state elicited brain activations in motor regions (primary motor cortex, supplementary motor area, premotor cortex, cerebellum), as well as additional areas (anterior cingulate, putamen, associative parietal areas); following L-dopa administration, the brain activation profile was globally reduced, highlighting activations in the parietal and posterior cingulate cortices. For the speech production task, brain activation patterns were similar with and without medication, including the orofacial primary motor cortex (M1), the primary somatosensory cortex and the cerebellar hemispheres bilaterally, as well as the left- premotor, anterior cingulate and supramarginal cortices. For the combined task *off* L-dopa, the cerebral activation profile was restricted to the right cerebellum (hand movement), reflecting the difficulty in performing two movements simultaneously in PD. Under L-dopa, the brain activation profile of the combined task involved a larger pattern, including additional fronto-parietal activations, without reaching the sum of the areas activated during the simple hand and speech tasks separately. Our results question both the role of the basal ganglia system in speech production and the modulation of task-dependent cerebral networks by dopaminergic treatment.

## Introduction

Studies on individuals with Parkinson’s disease (PD) have notably involved the investigation of the effects of dopaminergic medication on cerebral blood flow at rest [1]. Some studies reported no modifications of brain activation while others rather found a global increase [2], [3] or decrease [4], [5] in cerebral activity when comparing *on* medication *vs. off* medication state. Both subthalamic nucleus (STN) stimulation and levodopa (L-dopa) have proven to reduce hypermetabolism in the lenticular nucleus and increase metabolism in the associative prefrontal cortex [6]. Interestingly, the response to L-dopa at rest depends on the duration of exposure to the medication: individuals with PD chronically treated by L-dopa have decreased regional cerebral blood flow in the ventrolateral prefrontal and sensorimotor cortices, but drug-naive patients display no levodopa-induced modification of cerebral activation [7]. The significance of these changes in response to L-dopa remains uncertain but could reflect a modification of the thalamocortical projections by long-term L-dopa treatment, at least at rest.

Using self-generated arm movements in untreated individuals with PD, early functional neuroimaging studies reported reduced regional cerebral blood flow (rCBF) within the main cortical output areas of the basal ganglia, including the supplementary motor area (SMA), dorsolateral prefrontal cortex (DLPFC) and anterior cingulate cortex (ACG) [8]–[11]. These results were further extended in more recent studies that also revealed that the activation of some structures depends on the nature and complexity of the task. Thus, although the anterior SMA (pre-SMA) often appeared under-activated during hand movements, over-activations within the caudal SMA, premotor (PM), M1, inferior parietal and anterior cingulate cortices, as well as cerebellar hemispheres have also been reported, suggesting that these areas could be recruited to overcome the dysfunction of the cortex-basal-ganglia-cortex motor circuit [12]–[15]. In addition, the reduced activations reported within the SMA [9] and the lateral PM cortex [16] appeared to be partly restored following dopaminergic administration. Regarding M1 activation, the evidence on the effect of medication is contradictory. While some authors reported the restoration of activation after apomorphine administration [9], others showed a reduction of the precentral gyrus activation [17]. These contradictory findings could result from the specific constraints of the task being performed, while inhibition of endogenous dopamine release by the exogenous contribution of the medication may further complicate the matter [18]. Finally, the putamen and thalamus have been shown to be most responsive to levodopa, as compared with less responsive motor cortical areas [19]. Thus, these findings stressed the need to better understand the influence of dopaminergic treatments on the cortical and subcortical circuits [17]–[19] underlying movement production.

Motor network activations during PD speech production have been studied using both positron emission tomography (PET) [20]–[23] and functional magnetic resonance imaging (fMRI) [24]–[27]. These neuroimaging studies have documented that Parkinsonian speech seemed to be related to an altered recruitment of the main brain motor regions underlying speech production (orofacial motor cortex, cerebellum) and an increased involvement of the premotor and prefrontal cortices (DLPFC, SMA, superior premotor cortex). Additional cerebral activation, such as the recruitment of temporal regions, have also been observed *off* medication [24], [27], suggesting that a specific reorganization underlies the altered activation pattern associated with PD speech. In most PET experiments examining speech production in individuals with PD *off* or *on* treatment, basal ganglia activation barely reached statistical significance. A reduced SMA activation [21], [22], [25] and a significantly greater activation in the right primary orofacial sensorimotor cortex, as compared to controls subjects, have also been reported following L-dopa intake. These changes were interpreted as a compensatory phenomenon to preserve speech in PD [25]. They also revealed increased connectivity between the periaqueductal grey matter and basal ganglia, posterior superior temporal gyrus, supramarginal and fusiform gyri and inferior parietal lobule on the right side [26]. These modifications could reflect either a specific compensatory phenomenon or a specific modification of the activation pattern underlying brain dysfunctions of PD speech. In either case, this pattern of modifications does not parallel the one associated with hand motor tasks. Dysarthria usually worsens with disease progression, which suggests that it is also linked to the progression of the pathological processes to non-dopaminergic brain circuits [28]–[31]. Specifically, one would expect neuronal losses of additional areas of motor control to be involved, such as the pre-supplementary cortex [32], the thalamus [33] or the mesocortical system [34].

To date, no study has investigated the influence of dopaminergic treatment on the patterns of neural activation during hand and speech movements in the same group of individuals with PD. Furthermore, although in daily life speech is often accompanied by hand or other movements, very few studies have examined such dual tasking in PD [35]–[38]. A few neuroimaging studies have examined the performance of simultaneous movements in PD, as it is well-known that individuals with PD have difficulties performing complex, simultaneous or sequential movements [39]–[43]. Most of these studies have used bimanual movements [44], [45], or dual motor and cognitive tasks [46], rather than simultaneous limb and speech movements. While some of important issues regarding the neural bases of hand and speech movements in PD have been addressed, the functional brain activations underlying the difficulty these patients have in simultaneously performing both together remain unclear. Therefore, using fMRI, we investigated the influence of dopamine treatment on the patterns of neural activation underlying hand movement (HM) and speech production (SP) performed both alone and simultaneously in individuals with PD. Even if the (HM+SP) movement combination movement is unnatural and experimental, it allows for the combination of the two HM and SP movements: it should be considered as a combined task, and not a proper dual-task comprising strictly independent tasks, as it did not involve any cognitive conflict in response selection between the HM and SP tasks. The motor programming was identical for the two modalities, (*i.e.*, selection of the same response among four possibilities) and only the motor execution differed [24]. Moreover, it cannot be considered as a co-speech gesture also as it is not unconsciously self-generated by the participants themselves. We hypothesized that the brain activation profiles for HM and SP tasks performed alone would both be modulated following L-dopa intake. In a previous study, we observed that the brain activations recorded during the HM+SP combined task were the sum of the activations obtained when each of the tasks was performed separately in healthy subjects, while this was not the case in untreated individuals with PD [24]. Our second hypothesis was that L-dopa would restore such summation, as is the case for a bimanual simultaneous task [44].

## Materials and Methods

### 1. Patients

Twelve right-handed (Edinburgh handedness questionnaire >80%) patients with PD were recruited in the Neurological wards of the Grenoble (n = 6) and Lyon (n = 6) University Hospitals. Demographic and clinical characteristics of the patients are summarized in Table 1. All patients fulfilled the UK Parkinson’s disease Brain Bank Criteria [47] for the diagnosis of idiopathic PD and presented with predominant akinetic-rigid symptoms.

**Table 1:** Demographic data of the patients.

Patients	Sex	Symptom laterality predominance	Age (years)	Disease duration (years)
**1**	**M**	**R**	64	8
**2**	**M**	**L**	53	12
**3**	**M**	**R**	61	6
**4**	**M**	**R**	67	5
**5**	**M**	**R**	52	12
**6**	**M**	**L**	55	8
**7**	**M**	**L**	58	8
**8**	**M**	**L**	51	8
**9**	**M**	**L**	69	14
**10**	**F**	**L**	65	15
**11**	**M**	**L**	58	9
**12**	**M**	**L**	64	12
		**Mean** ± SD	**59.8**±6.1	**9.8**±3.2

F = female; L = left; M = male; R = right; SD = Standard deviation.

All patients were studied first without (*off*), and then with (*on*) anti-Parkinsonian medication, during two consecutive fMRI sessions of one hour each. *Off* medication, patients were scanned after an overnight fast, *i.e.* at least 12 hours of PD treatment withdrawal. The *on* medication session was undertaken with patients in their *best on* motor state, 45 to 60 minutes after administration of a suprathreshold dose of L-dopa (120% of the usual morning dose; see Table 2). Unblinded evaluation of the patients’ global motor disability was performed before each fMRI session using the motor section of the Unified Parkinson’s Disease Rating Scale (UPDRS, part III) [48]. Only patients with mild to moderate mostly akineto-rigid symptoms (with no or little tremor *off*, and no or little dyskinesia *on*) were included to ensure that they could perform the tasks. The patients had moderate speech impairment and were able to produce intelligible speech allowing for the performance of the speech production task. The *off* and *on* medication UPDRS scores were statistically compared (paired Student t test, p<0.05; STATISTICA 7.1, Statsoft, Tulsa, USA) to appreciate the impact of the treatment.

**Table 2:** Clinical data of the patients.

Patients	Hoen & Yahr scale (score/5)		Total UPDRS motor score, items 18–31, score/108		Speech, item 18, score/4		Tremor, items 20–21, score/28		Rigidity, item 22, score/20		Axial signs, items 18, 22 (neck), 27–30, score/40		Akinesia, items 23–26, score/32		L-dopa equivalent dose (mg)
	off	on	off	on	off	on	off	on	off	on	off	on	off	on	
**1**	3	1	38	9	1	1	8	1	7	5	12	6	15	2	400
**2**	3	2.5	40	9	1	1	5	0	14	2	18	4	14	5	155
**3**	3.5	2	36	7	1	0	1	1	10	2	16	2	16	3	350
**4**	3	2	49	10	1	1	6	0	10	4	16	4	24	6	300
**5**	3.5	2	43	19	2	1	9	1	11	6	17	9	12	6	300
**6**	2	1	32	5	1	0	3	0	6	1	14	3	11	1	400
**7**	3	2	51	16	2	1	6	1	12	5	20	7	21	6	250
**8**	4	2	37	3	1	0	1	0	4	0	13	0	18	2	350
**9**	4	2.5	39	6	1	1	1	0	9	2	20	5	15	0	225
**10**	3	2	42	18	2	1	1	0	11	6	21	9	16	7	350
**11**	3.5	2.5	40	8	1	1	7	0	11	3	17	5	13	2	200
**12**	3	2	36	10	1	1	2	1	6	1	15	4	14	5	350
**Mean ± SD**	**3.1±0.6**	**1.8±0.4**	**40.3±5.4**	**10.0±5.1**	**1.3±0.5**	**0.8±0.5**	**4.2±3.0**	**0.4±0.5**	**9.3±2.9**	**3.1±2.1**	**16.6±2.8**	**4.8±2.7**	**15.8±3.7**	**3.8±2.3**	**303±79**

All *off* and *on* medication motor scores were obtained using of the motor section of the UPDRS [48]. The UPDRS is a five-point scale, meaning that the scoring range for each item spans from “0" ( = normal) to “4" ( = maximal impairment). For example, speech can be assessed with item 18 using the following scores: “0" = normal; “1" = slight loss of expression, diction and/or volume; “2" = monotone, slurred but understandable (moderately impaired); “3" = marked impairment, difficult to understand; “4" = unintelligible. SD = Standard deviation.

### 2. Ethics Statement

The study (project n° 06-CHUG-6) was conducted in accordance with the Declaration of Helsinki [49], approved by the local Ethics Committee Review Board (Comité de Protection des Personnes [CPP] pour la recherche biomédicale, Centre Hospitalier Universitaire de Grenoble [CHUG], France). The patients were included after providing written informed consent.

### 3. Experimental Paradigms

The experimental protocol included 3 motor tasks [24]:

*Hand movement (HM)* - a freely chosen sequence of movements performed with the right hand, moving a non-magnetic fMRI-compatible joystick (Current Designs, Philadelphia, USA) in 4 possible directions (forward, backward, right and left), starting from and returning to the neutral position;
*Speech production (SP)* - a freely chosen speech sequence, using 4 possible words (“Up", “Down", “Right" and “Left");
*Combined task ([HM+SP])* - a freely chosen sequence of joystick movements performed with the right hand together with the speech description of the associated directions (“Up", for the forward direction; “Down", for the backward direction; “Right"; and “Left").

They represented a particular version of externally-paced tasks, since an additional dimension was introduced by the choice among the 4 possibilities. Prior to the fMRI trials, patients had ample opportunity to practice the tasks, during which they were instructed to respond to each visual stimulus, *i.e.* the word “action" presented every two seconds for 500 milliseconds (ms). Patients were asked to avoid pre-established sequences of movement directions. They were allowed to use the same direction and/or produce the same word several times in a row. The joystick used for the HM and (HM+SP) tasks was connected to a control case enabling the synchronization of the visual stimulus with image acquisition. The setting also enabled *a posteriori* monitoring of performance, as well as analysis of the direction of the movements. During a rest condition (the word “rest" appeared repeatedly on the screen), patients were required to remain still, without making any movement or speaking. For technical reasons, including the noisy fMRI environment, we were not able to monitor the SP task performance. Nevertheless, *a posteriori* questioning of the patients ensured that the tasks had been performed according to the instructions.

### 4. Functional MRI Procedure

To ensure patients’ comfort, a block-design was chosen, each motor task corresponding to an fMRI run of about 7 minutes’ duration. Each run alternated 10 blocks of rest and 10 blocks of action (HM, SP or tasks HM+SP), each block including 10 trials, for a total of 100 trials. The order of the fMRI runs was counterbalanced between conditions and patients. fMRI data were obtained on a 3 Tesla (T) MRI scanner (Bruker Medspec S300 - IFR 1, Biomedical NMR and Neurosciences, Grenoble, France), equipped with an emitting/receiving head coil. For each fMRI run, 160 volumes covering the whole brain with 40 adjacent axial 3.2 mm thick slices were acquired using a BOLD-contrast multi-slice T2*-weighted single-shot echo-planar imaging (EPI) sequence (echo time (TE) = 30 ms, repetition time (TR) = 2500 ms, flip angle = 77°, field of view (FOV) = 216×216 mm^2^, matrix size 72×72, voxel size = 3×3×3.2 mm^3^). T1-weighted 3D magnetization prepared rapid acquisition gradient echo (MP-RAGE) anatomical images of the whole brain were also acquired (TR = 2500 ms, TE = 3.89 ms, TI = 900 ms, flip angle = 8°, FOV = 256×224×176 mm^3^, voxel size = 1.33×1.75×1.375 mm^3^).

### 5. Behavioral Data Analysis

Direction and response times (RT) for the HM and (HM+SP) tasks were recorded during image acquisition and processed *off* line. RTs shorter than 150 ms or longer than 1500 ms were discarded. Mean RT and performance rate were computed for each patient. The influence of task (single *vs.* double) and medication (*off vs. on*) was analyzed using non-parametric Wilcoxon tests with a corrected p-value<0.025 for multiple comparisons.

### 6. fMRI Data Analysis

fMRI data analysis was performed using SPM5 software (Wellcome Department for Cognitive Neuroscience, London, UK) [49]. First-level analyses were carried out for each patient, yielding parametric statistical maps generated for each motor task both *off* and *on* medication. Patient 4 was not able to perform the SP task *off* medication and patient 7 the HM task *on* medication. The first-level contrasts were introduced within second-level analyses, using a two-factor ANOVA model with the motor tasks (HM, SP and HM+SP) and treatment conditions (*off* or *on* medication) as repeated measures. Only cerebral areas whose statistical thresholds corresponded to probabilities p_FWE-corrected_<0,05 at the voxel level and activation foci for which the number of voxels was equal or superior to k = 10 were retained (Z-scores>5.20). When this statistical processing did not make it possible to detect any activation, a non-corrected statistical threshold of p_uncorrected_<0,001 was applied at the voxel level (Z-scores>3.10). The activation coordinates were transformed into a standard stereotactic space [50]. Between-medication state and between-task comparisons allowed for further detailed analysis. Uncorrected p-values<0.001 (Z-scores>3.10) were considered significant for these contrasts.

## Results

### 1. Clinical and Behavioral Data

Group analysis confirmed that the total motor scores of the UPDRS and the Hoen and Yahr scale (Table 2) decreased significantly following the administration of L-dopa (75.2% of improvement for the UPDRS, p<0.001; 41.9% of improvement for the Hoen and Yahr, p<0.001). The improvement for rigidity, axial signs and akinesia sub-scores was 66.7%, 71.1% and 75.9% respectively (p<0.001). The improvement in speech production as assessed using item 18 of the UPDRS III was also significant, although more limited (38.5%, p<0.001).

The patients correctly performed the tasks under the two experimental conditions, although the number of movements was greater *on* medication than *off* (HM *off*: 62±18; *on*: 78±15; HM+SP *off*: 64±18; *on*: 78±12). The effect of medication on the number of trials was significant both for the HM task (z = 2.85; p<0.005) and for the combined task (z = 2.90; p<0.005). There was no effect of task on the number of movements. Regarding response time, there was no effect of either task or medication, although response times were slightly shorter under medication than *off* (HM *off*: 659±173 ms; *on*: 556±101 ms; HM+SP *off*: 626±145 ms; *on*: 561±116 ms). Neither task nor medication affected the distribution of the movements’ direction.

### 2. fMRI within-group Comparisons and between Medication State Comparisons

#### 2.1. Hand movement

**Figure 1:**
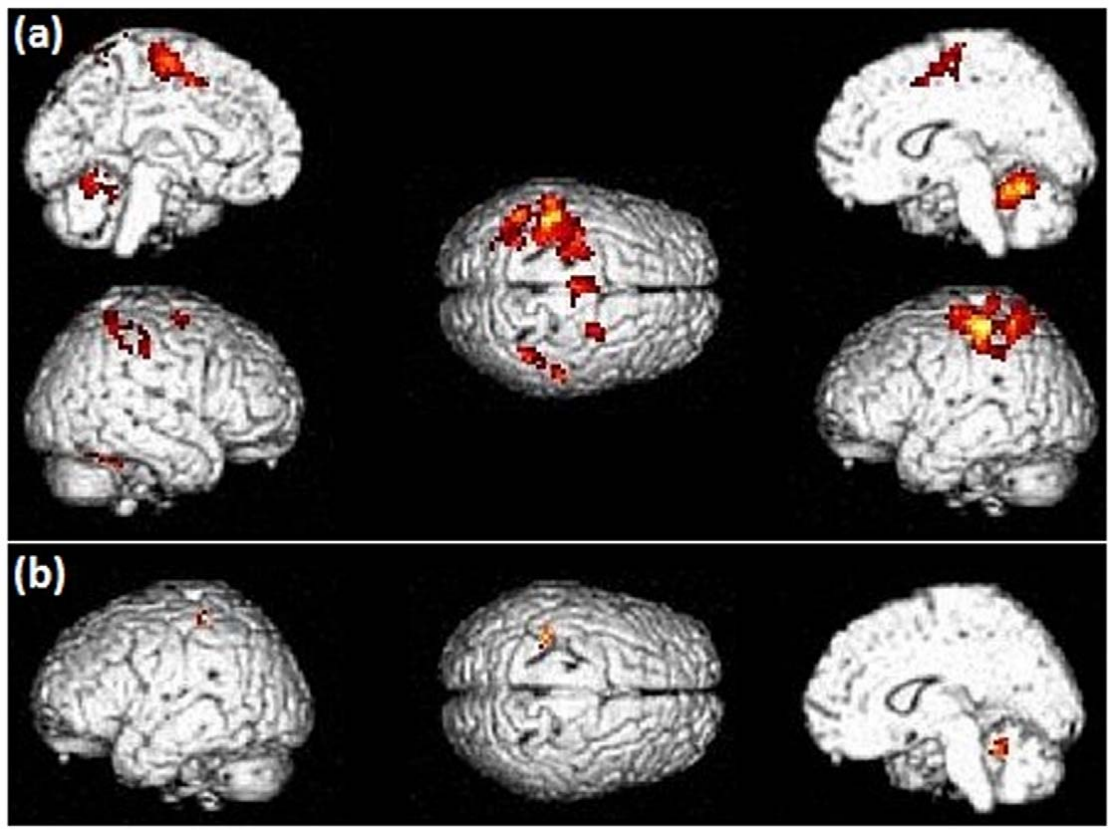
Patterns of brain activation during the hand movement task in PD patients (a – *off* L-dopa; b – *on* L-dopa). Activation thresholds correspond to corrected (FWE) p-values<0.05. Without medication, brain activations were found in the right cerebellum, left motor/premotor regions, anterior cingulate cortex, superior and inferior parietal lobules, and putamen; following L-dopa intake, the brain activation profile was globally reduced, restricted to weak activations in the right posterior cingulate gyrus and the left inferior parietal lobule.

At a comparable statistical level (Table 3), the brain activation profile *off* L-dopa strongly involved the right cerebellum and left motor/premotor regions; recruitment of the anterior cingulate cortex and the superior and inferior parietal lobules were also noted, as well as that of the putamen (Fig. 1a). Administration of L-dopa led to a focalization of activations, revealing weak activations in the right posterior cingulate gyrus and the left inferior parietal lobule (Fig. 1b). Cerebral activations in the *off vs. on* L-dopa comparison involved significant right-lateralized regions in the anterior insula and the putamen; for the *on vs. off* L-dopa comparison, no suprathreshold clusters were found for the HM task (Table 4).

**Table 3:** Cerebral sites of maximal hemodynamic responses during the hand movement (HM): Main effects (p_FWE-corrected_<0.05, k≥10) of L-dopa medication states.

			off L-dopa					on L-dopa				
Cerebral area	BA	L/R	x	y	z	Z-score	k	x	y	z	Z-score	k
**Precentral gyrus**	4	L	−19	−27	56	6.12	521°					
**Medial frontal gyrus**	6	L	−3	−18	52	5.95	151^#^					
**Frontal sub-gyral**	6	R	23	−6	52	5.51	29					
**Anterior cingulate gyrus**	24	L	−1	0	45	5.62	151^#^					
**Posterior cingulate gyrus**	31	R						18	−48	−24	5.26	20
**Superior parietal lobule**	7	R	34	−53	52	5.20	64^∧^					
**Inferior parietal lobule**	40	L	−40	−53	47	6.77	521°	−30	−38	51	5.63	12
Inferior parietal lobule	40	R	31	−44	37	5.88	64^∧^					
Inferior parietal lobule	40	R	47	−30	38	5.56	28					
**Putamen**		L	−27	−12	4	5.29	14					
**Cerebellum (dentate)**		L	−14	−50	−24	5.42	18					
Cerebellum (dentate)		R	15	−50	−21	7.61	251*					
**Cerebellum (vermis)**		R	2	−62	−14	6.19	251*					
**Cerebellum (hemisphere)**		R	26	−64	−25	6.09	251*					

Cerebral activation locations refer to maximal hemodynamic response sites. L/R: left/right; BA: Brodmann’s area; x, y, z: mediolateral, rostrocaudal and dorsoventral Talairach coordinates; k: cluster size (number of voxels); °/^#^/^∧^/*: parts of the same cluster.

**Table 4:** Cerebral sites of maximal hemodynamic responses during the hand movement (HM): Between medication state comparisons (p_uncorrected_<0.001, k≥10).

		off *vs.* on L-dopa					on *vs.* off L-dopa				
Cerebral area	L/R	x	y	z	Z-score	k	x	y	z	Z-score	k
**Insula (anterior)**	R	34	8	−5	3.66	19	*No suprathreshold clusters*				
**Putamen**	R	26	8	0	3.28	19					

Cerebral activation locations refer to maximal hemodynamic response sites. L/R: left/right; BA: Brodmann’s area; x, y, z: mediolateral, rostrocaudal and dorsoventral Talairach coordinates; k: cluster size (number of voxels).

#### 2.2. Speech production

**Figure 2:**
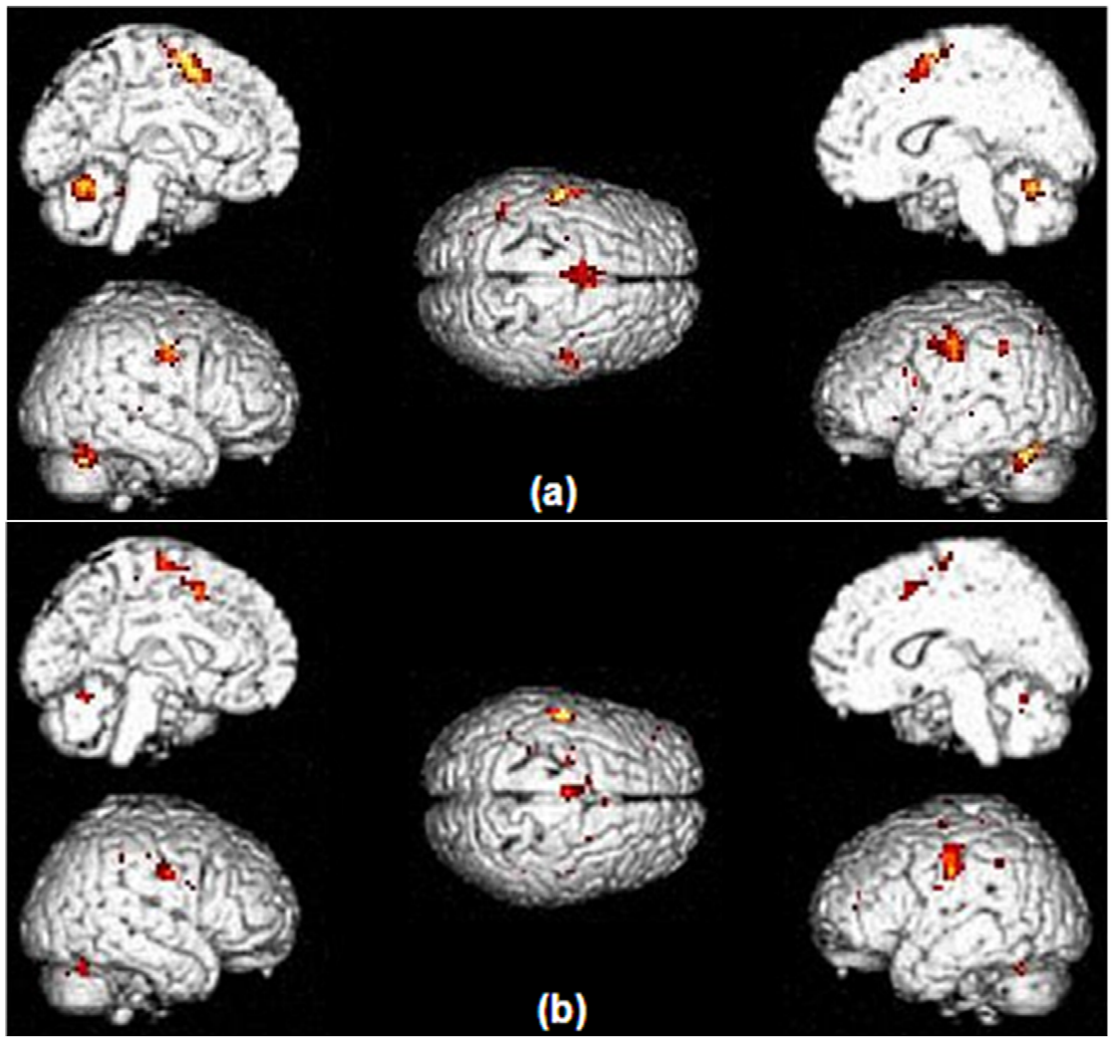
Patterns of brain activation during the speech production task in PD patients (a – *off* L-dopa; b – *on* L-dopa). Activation thresholds correspond to uncorrected p-values<0.001. Brain activation patterns were similar with or without medication, including the bilateral orofacial M1 and cerebellar hemispheres, as well as the left-sided premotor, primary somatosensory, supramarginal and anterior cingulate cortices.

No significant brain activations survived the p_FWE-corrected_<0.05 statistical level. Values at p_uncorrected_<0.001 (Table 5) revealed similar activations for the *off* and *on* L-dopa conditions, including the bilateral orofacial M1 and cerebellar hemispheres, as well as the left premotor, primary somatosensory, supramarginal and anterior cingulate cortices,. In terms of cluster sizes, the *off* L-dopa (Figure 2a) brain activation profile was larger than *on* L-dopa (Figure 2b) at the levels of premotor and anterior cingulate cortices, and cerebellum. For the SP task, no suprathreshold clusters were detected either for *off vs. on* L-dopa or o*n versus off* L-dopa.

**Table 5:** Cerebral sites of maximal hemodynamic responses during speech production (SP): Main effects (p_uncorrected_<0.001, k≥10) of L-dopa medication states.

			off L-dopa					on L-dopa				
Cerebral area	BA	L/R	x	y	z	Z-score	k	x	y	z	Z-score	k
**Precentral gyrus**	4	L	−46	−15	39	4.06	95°	−46	−15	39	4.29	94^∧^
Precentral gyrus	4	R	41	−12	33	4.67	58	41	−12	28	4.09	36*
**Medial frontal gyrus**	6	L	−6	−6	55	4.97	123^#^	−3	−15	57	3.97	27
**Postcentral gyrus**	2	L	−35	−21	33	4.40	95°	−38	−21	28	4.32	94^∧^
Postcentral gyrus	2	R						36	−18	30	4.07	36*
**Anterior cingulate gyrus**	24	L	−6	0	47	4.63	123^#^	−6	5	42	3.94	33
**Supramarginal gyrus**	40	L	−40	−47	32	3.71	33	−11	−64	−19	3.73	10
**Cerebellum (dentate)**		L						−11	−64	−19	3.73	10
**Cerebellum (hemisphere)**		L	−25	−62	−27	5.79	149	−27	−62	−27	4.01	10
Cerebellum (hemisphere)		R	26	−64	−25	5.25	118	26	−64	−25	4.07	17

Cerebral activation locations refer to maximal hemodynamic response sites. L/R: left/right; BA: Brodmann’s area; x, y, z: mediolateral, rostrocaudal and dorsoventral Talairach coordinates; k: cluster size (number of voxels); °/^#^/^∧^/* : parts of the same cluster.

#### 2.3. Combined task

**Figure 3:**
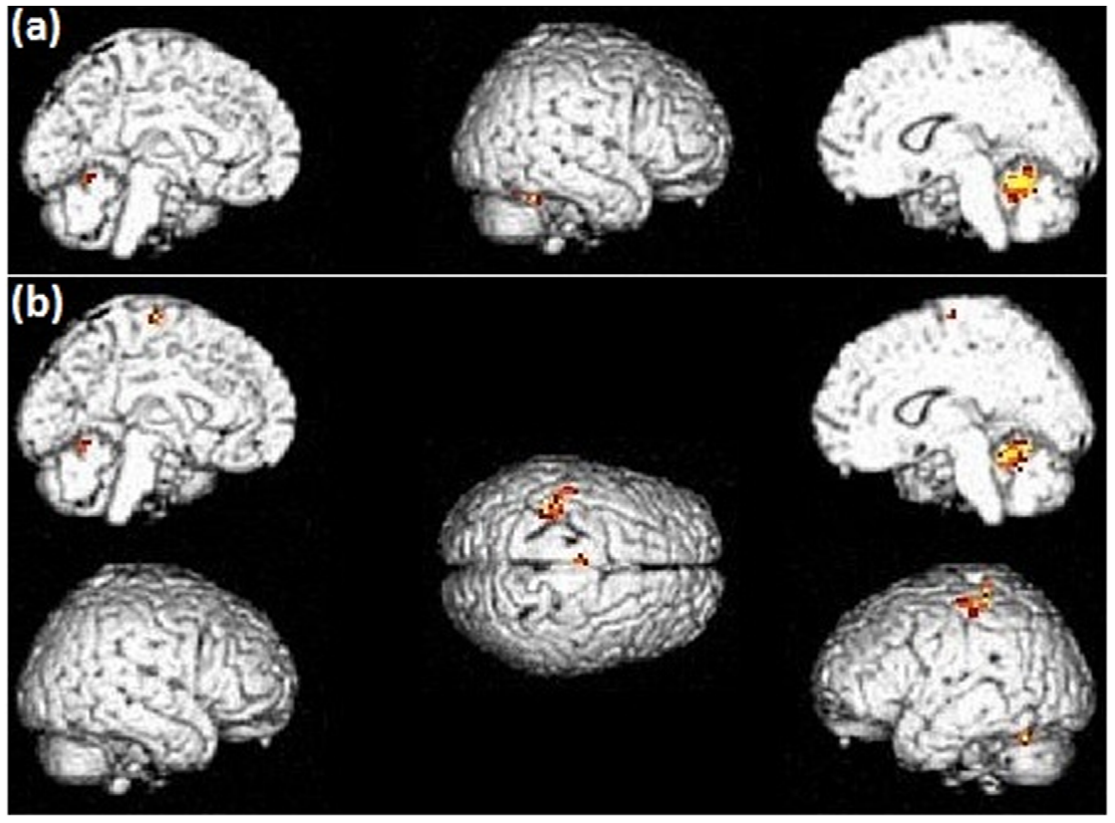
Patterns of brain activation during the [hand movement + speech production] task in PD patients (a – *off* L-dopa; b – *on* L-dopa). Activation thresholds correspond to corrected (FWE) p-values<0.05. Without L-dopa, the combined task yielded a right cerebellar activation. Under L-dopa, comparable cerebellar activations were seen, along with additional left-sided activations in the medial premotor cortex, post-central gyrus and inferior parietal lobule.

Activations within the right cerebellum were seen *off* L-dopa (Fig. 3a). *On* L-dopa, comparable cerebellar activations were seen (Table 6), although there was a focalization of the areas recruited (Fig. 3b). Additional left-sided activations were seen in the medial premotor cortex, the post-central gyrus and the inferior parietal lobule. *Off versus on* L-dopa comparison for the (HM+SP) task revealed an activation within the right median temporal gyrus. No suprathreshold clusters were detected *on versus off* L-dopa (Table 7).

**Table 6:** Cerebral sites of maximal hemodynamic responses during the combined [HM+SP] task: Main effects (p_FWE-corrected_<0.05, k≥10) of L-dopa medication states.

			off L-dopa					on L-dopa				
Cerebral area	BA	L/R	x	y	z	Z-score	k	x	y	z	Z-score	k
**Medial frontal gyrus**	6	L						−3	18	60	5.30	10
**Postcentral gyrus**	3	L						−30	−32	43	5.27	43^#^
Postcentral gyrus	40	L						−40	−30	51	5.07	43^#^
**Inferior parietal lobule**	40	L						−30	−38	51	5.04	43^#^
**Cerebellum (dentate)**		R	12	−56	−19	5.87	94*	15	−50	−21	5.60	71
**Cerebellum (hemisphere)**		L						−27	−62	−27	5.68	11
Cerebellum (hemisphere)		R	26	−64	−25	5.84	94*					

Cerebral activation locations refer to maximal hemodynamic response sites. L/R: left/right; BA: Brodmann’s area; x, y, z: mediolateral, rostrocaudal and dorsoventral Talairach coordinates; k: cluster size (number of voxels); *, ^#^: parts of the same cluster.

**Table 7:** Cerebral sites of maximal hemodynamic responses during the combined [HM+SP] task: Between medication condition comparisons (p_uncorrected_<0.001, k≥10).

			off *vs.* on L-dopa					on *vs.* off L-dopa				
Cerebral area	BA	L/R	x	y	z	Z-score	k	x	y	z	Z-score	k
**Temporal sub-gyral**	21	R	47	−9	−9	3.94	28	*No suprathreshold clusters*				

Cerebral activation locations refer to maximal hemodynamic response sites. L/R: left/right; BA: Brodmann’s area; x, y, z: mediolateral, rostrocaudal and dorsoventral Talairach coordinates; k: cluster size (number of voxels).

### 3. fMRI between-task Comparisons

*Off* L-dopa, no (HM+SP) *vs.* HM task activations were detected; *on* L-dopa, there was a right-sided M1 activation (Table 8). *Off* L-dopa, the (HM+SP) *vs.* SP contrast was associated with left activations within the cerebellum, premotor cortex, superior temporal gyrus, as well as superior and inferior parietal lobules. *On* L-dopa, analyses revealed no suprathreshold clusters (Table 9).

**Table 8:** Cerebral sites of maximal hemodynamic responses highlighted by between-task comparisons under off and on L-dopa conditions: Combined (HM+SP) task *vs*. Hand movement (HM) task (p_uncorrected_<0.001).

			off L-dopa					on L-dopa				
Cerebral area	BA	L/R	x	y	z	Z-score	k	x	y	z	Z-score	k
**Precentral gyrus**	4	R	*No suprathreshold clusters*					41	−15	46	4.06	18

Cerebral activation locations refer to maximal hemodynamic response sites. L/R: left/right; BA: Brodmann’s area; x, y, z: mediolateral, rostrocaudal and dorsoventral Talairach coordinates; k: cluster size (number of voxels).

**Table 9:** Cerebral sites of maximal hemodynamic responses highlighted by between-task comparisons under off and on L-dopa conditions: Combined (HM+SP) task *vs.* Speech production (SP) task (p_uncorrected_<0.001).

			off L-dopa					on L-dopa				
Cerebral area	BA	L/R	x	y	z	Z-score	k	x	y	z	Z-score	k
**Paracentral lobule**	31	L	−3	−18	44	3.85	11	*No suprathreshold clusters*				
**Superior temporal gyrus**	41	L	−40	−32	17	4.14	16					
**Superior parietal lobule**	7	L	−27	−56	58	4.06	177*					
**Inferior parietal lobule**	40	L	−43	−35	48	4.41	177*					
**Postcentral gyrus**	40	R	52	−24	17	3.64	11					
**Cerebellum (dentate)**		R	18	−50	−24	4.54	67					

Cerebral activation locations refer to maximal hemodynamic response sites. L/R: left/right; BA: Brodmann’s area; x, y, z: mediolateral, rostrocaudal and dorsoventral Talairach coordinates; k: cluster size (number of voxels);

* parts of the same cluster.

## Discussion

*Off* medication, unilateral hand movements yielded brain activations in the right cerebellum, left motor/premotor regions, anterior cingulate cortex, superior and inferior parietal lobules, as well as in the putamen. Under L-dopa, the brain activation profile was globally reduced, restricted to activations in the right posterior cingulate gyrus and the left inferior parietal lobule. For the speech production task, brain activation patterns were similar with and without medication, including the bilateral orofacial M1 and cerebellar hemispheres, as well as the premotor, primary somatosensory, supramarginal and anterior cingulate cortices, all left-sided. The combined task yielded a right cerebellar activation, both without and under L-dopa. *On* L-dopa, there were additional left-sided activations in the medial premotor cortex, post-central gyrus and inferior parietal lobule.

### 1. Hand Movements in PD

Behavioral measurements showed an improvement in the ability to respond to commands and initiate the hand movement following L-dopa intake, as evidenced by the increased number of movements. The shortening in response time did not reach significance, which is not surprising as, unlike other studies that reported an effect of medication on reaction time [51], [52], we did not use a reaction time protocol. The reduced performance of hand movements when *off* L-dopa could be related to the overactivation of the cortico-cerebello-cortical circuit and the loss of brain activation selectivity resulting from the effort to overcome the dopaminergic-denervation-dependent akinesia [12], [14]. Several neuroimaging studies supported the hypothesis that an increased activation of the cortico-cerebello-cortical circuitry may compensate for the cortico-striato-cortical motor loop dysfunction [12]–[15], [53], [54]. Despite the stronger recruitment of the cerebellum in the *off* medication brain profile and the reductions in brain activation following L-dopa intake, the between-medication state comparison did not reveal any cerebellar over-activation in the present study. Other authors also failed to observe this cerebellar over-activation in individuals with PD, *off* medication [55]–[57], including one using the same experimental paradigms [24]. Such discrepancies may depend on whether the brain activation profile of PD patients’ *off* medication is compared with the *on* medication state or with controls subjects, highlighting the fact that medication does not restore a normalized pattern.

The strong fronto-parietal involvement *off* L-dopa included the inferior and superior parietal gyri (BA 40 and 7), with predominance in the left hemisphere. In contrast to earlier findings [24], there was no associated visual activation, although the pacing stimuli were presented visually. Under medication, the brain activation profile, including the parietal network, was globally reduced, although a left-sided activation persisted within the inferior parietal lobule. It has been shown that improved movement performance is associated with a reduction of brain compensatory activations following L-dopa or subthalamic nucleus stimulation, that is, after restoration of the more efficient cortico-striato-thalamo-cortical pathway [16], [18], [58], [59]. Our results suggest that although dopaminergic administration can improve motor performances in PD patients, it does so through a reorganized dopaminergic system: the cerebellar and parietal implications *off* medication were reduced following L-dopa administration. Nevertheless, the only regions surviving the between medication condition comparisons were found in the right anterior insula and putamen.

### 2. Preservation of Speech in PD

In both *off* and *on* medication conditions, we found premotor/motor brain activations in the frontal cortex and cerebellum similar those previously reported [20]–[27]; activations within the left anterior cingulate cortex and supramarginal gyrus were also observed. These regions could be part of a fronto-parietal compensatory mechanism enabling the preservation of speech in PD [24], [27]. Indeed, the speech production task was likely driven by the cognitive decision of the direction rather than internal vocalization, as no associative auditory activation was found. As observed for the HM single task, an overall reduction in brain activations was observed, being much more important in the cerebellum and the premotor, anterior cingulate and supramarginal cortices, than in the motor regions. Orofacial activity seemed to respond somewhat like hand activity, albeit to a lesser extent. Others have reported a right orofacial sensorimotor cortex compensatory activation in PD patients *on* medication when compared to control subjects [25]. Lateralisation of basal ganglia dysfunction in PD has also to be taken into account, since evidence of “a crucial role of the right basal ganglia in the maintenance of isochronous speech rhythms" has been recently reported when comparing performances of two homogenous groups of PD patients, presenting with right and left-sided predominant symptoms [60].

A key connection between the basal ganglia and the cerebellar motor circuits seems to be altered in PD and a reduction of brain activation within both circuitries may participate in the development of dysarthria. Our data suggest that compensatory strategies could involve, amongst others, temporal regions which are known to play a role in SP mechanisms, the insula, and frontal areas including the lateral premotor cortex and the anterior cingulate gyrus (ACG). All these regions could be part of a surrogate network able to provide the functionality needed to support SP, even if SP is dysarthric. Among these regions, the ACG is one of the main basal ganglia circuit outputs (the limbic one). On the other hand, the cerebellum is tightly connected with frontal regions, including Broca’s area and the lateral premotor cortex. The additional network activated in our patients could therefore be recruited as compensatory circuitry of dysarthric speech, enabling inter-circuit compensations, either in the fronto-striatal or the cerebro-cerebellar circuitries. It is possible that in early PD, basal ganglia dysfunction primarily affects speech and leads to mild dysarthria, while with the disease progression severe dysarthria results from additional dysfunction of the cortex-cerebellum circuitry. It is known that worsening of dysarthria with disease progression parallels an increasing severity of non-dopaminergic cerebral lesions [28], [29], [31]. One key structure could be the thalamus [33], since it relays to the cortex information arising in both the cerebellum and the basal ganglia loops.

The key-role of the cerebellum [61]–[63] and non-dopaminergic neural circuits in the anatomo-functional substrate of vocal communication could partly explain the resistance of Parkinsonian speech to L-dopa. Even if some studies pointed out beneficial effects of L-dopa on PD speech [64], [65], several studies that have explored speech parameters in PD have demonstrated the lack of significant change between the two medication condition [66]. According to the classical cortico-subcortical circuit models first defined in the early nineties [67]–[69], and further developed since [70], [71], the circuits operate in a segregated and parallel fashion. The concept of closed and open circuits allows for cross-communication between circuits [72]. Apart from the motor circuit, which connects the primary motor/lateral premotor cortices and the SMA via the putamen, parallel non-motor loops originate from various regions of the basal ganglia and terminate in the DLPFC (prefrontal loop), orbito-frontal cortex (lateral orbito-frontal loop) or even anterior cingulate cortex (limbic loop). It is therefore possible that the alteration of the motor loop be compensated for, albeit imperfectly, by the recruitment of a non-motor circuit. The motor deficit of PD speech could depend on such a compensatory pathway: perceptual evaluation through the UPDRS speech item demonstrated that the patients presented with mild dysarthria, whether *on* or *off* medication. Indeed, clinical data revealed a significant improvement of the UPDRS motor scores under medication, but as already reported, improvement was lesser for speech production [73], [74]. Our results regarding the speech production task in PD are thus consistent with the idea that the recruitment of non-dopamine dependent cerebral compensatory mechanisms allows for acceptable speech performance.

### 3. Combining Movements in PD

Studies using dual-task paradigms combining simultaneous but independent verbal and manual tasks are scarce [75]–[77], including in PD patients [35]–[38]. Yet, such concurrent tasks are common in daily activities and their performance is often impaired in PD [78]. The combined task was specifically formulated to avoid any cognitive conﬂict; this was an advantage for assessing the neural correlates of simultaneous task performance, without cognitive overload, which is usually a confounding factor in dual-task paradigms. The combined task paradigm was not a classical dual task one, as it did not involve strictly independent tasks, that is, it did not induce cognitive conﬂict in response selection between the HM and SP tasks. For both HM and SP, the motor processes yielded the selection and planning of the same response (left, right, up, down) among four possibilities (*i.e.* the four movement directions). Only the motor execution (hand and orofacial movements) differed in terms of the muscles involved in production. Thus, PD patients did not face any conflict, but rather facilitation, in response selection during the task. PD individuals have been shown to demonstrate trouble when performing complex dual tasks and exhibit greater activations in the cerebellum, premotor area, precuneus, parietal and prefrontal cortices [46] than control subjects. In a previous study, in control subjects, but not in PD patients *off* medication, our combined task yielded the sum of the brain activations obtained during hand movement and speech production performed separately [24]. This was interpreted as the patients’ functional prioritization of the HM, SP being therefore associated with sub-threshold cerebral activations. It could also have reflected the inability of PD patients to intrinsically engage in the motor coordination necessary to perform a combined task. Indeed, due to the additive nature of the two tasks, the combined task represented an easier task to generate.

In the present study, our data are in agreement with those observed previously in patients *off* medication. Moreover, the summation of the two networks was also absent when the patients were *on* medication. This is congruent with the fact that SP activations never reached the corrected statistical threshold, even during the performance of the single task. These results suggest that the loss of capacity-sharing in combined movements did not improve with L-dopa, contrary to our *a priori* hypothesis. In fact, weak activations, restricted to the single right cerebellum, were revealed *off* L-dopa; this lateralization suggested that it was related to the hand movement part of the combined task. Following L-dopa administration, left-sided regions were activated in a premotor fronto-parietal network. Activations of these regions were also seen during the single hand movements, and interpreted as possible compensatory recruitments. These regions, possibly involved in compensatory pathways, were highlighted by the between-task comparisons (Tables 8 and 9). Thus, unlike what was seen for the single tasks performed separately, this activation pattern suggests that PD patients may rely on this network for simultaneous motor performance [14], [79], [80]. Palmer *et al.* [44] showed that during bimanual movements in PD, L-dopa partially normalized the effective connectivity and temporal patterns of activity. As already stressed by Brown [81], the authors related the patients’ inability to perform two movements simultaneously to the impaired capacity of binding the widespread cortical and subcortical areas underlying dual-task performance. They suggested that L-dopa restored effective communication between these areas, and/or reduced “the excessive beta-band synchronization that permeates widespread areas in the cortex and basal ganglia" (pages 701–702). Our results do not support the idea that L-dopa can restore coordinated cortical/sub-cortical recruitments in dual-tasks, although limb motor function appeared to be more dopamine-driven (HM) than speech.

### 4. Methodological Issues

Individuals with PD patients underwent the fMRI experiment without anti-Parkinsonian medication. Since the *off* medication state was evaluated in the morning of the experiment, and in order to minimize any differential effects across conditions, we counterbalanced the order of the experimental conditions across subjects. The patients who participated in this study represented a homogenous group of PD patients and may not reflect the range or variability of the disease. They were all relatively young patients, most of them in accordance with the required inclusion criteria for deep brain stimulation. Under the *off* medication condition, the patients did not have any tremor. They were predominantly akinetic-rigid, and one could wonder what would have been our findings with tremor predominant patients with PD. It is hard to anticipate whether the same findings would be obtained in such a sub-group of patients; to our knowledge, no distinction has been suggested so far regarding speech pathophysiology of akinetic-rigid *vs.* tremor PD patients. All patients were producing intelligible speech (*cf.* Table 2; mean UPDRS item 18 speech score = 1.3±0.5; the worst score being 4) and unfortunately, we were not able to record speech production either inside or outside the fMRI scanner: we acknowledge that another clinical measure for speech production should have provided a differentiated picture of the patients’ potential deficit in this domain. Altogether, these methodological aspects have to be taken into account when interpreting the results, possibly restricting generalization.

### Conclusions

Our results question both the role of the basal ganglia system in speech production and the modulation of task-dependent cerebral networks by dopaminergic treatment. Whereas the hand movement brain network is sensitive to dopaminergic medication, the brain activation patterns of speech production appear to undergo little changes following medication. Even if different compensatory circuits are activated in PD to try and overcome difficulties in performing hand and speech movements, notably temporal regions for speech production, recruitment of the associative parietal cortex seems to be an alternative in tasks sharing programming modalities. While conflicting dual task might result in preferential execution of one of the two tasks performed simultaneously, the combined task we used in this study did not lead to positive synergistic effect under the *on* medication state. Further experiments enabling the concomitant recording of speech and hand movement, both conflicting and synergistic, are required to confirm such finding.

## References

[1] Brooks DJ (2010) Imaging approaches to Parkinson disease. J Nucl Med 51: 596–609. 20351351 10.2967/jnumed.108.059998

[2] Leenders KL, Wolfson L, Gibbs JM, Wise RJ, Causon R, et al (1985) The effects of L-DOPA on regional cerebral blood flow and oxygen metabolism in patients with Parkinson’s disease. Brain 108 (Pt 1): 171–191. 10.1093/brain/108.1.171 3978397

[3] Montastruc JL, Celsis P, Agniel A, Demonet JF, Doyon B, et al (1987) Levodopa-induced regional cerebral blood flow changes in normal volunteers and patients with Parkinson’s disease. Lack of correlation with clinical or neuropsychological improvements. Mov Disord 2: 279–289. 3509776 10.1002/mds.870020405

[4] Berding G, Odin P, Brooks DJ, Nikkhah G, Matthies C, et al (2001) Resting regional cerebral glucose metabolism in advanced Parkinson’s disease studied in the off and on conditions with [(18)F]FDG-PET. Mov Disord 16: 1014–1022. 11748732 10.1002/mds.1212

[5] Gotham AM, Brown RG, Marsden CD (1988) ‘Frontal’ cognitive function in patients with Parkinson’s disease ‘on’ and ‘off’ levodopa. Brain 111 (Pt 2): 299–321. 10.1093/brain/111.2.299 3378138

[6] Hilker R, Voges J, Thiel A, Ghaemi M, Herholz K, et al (2002) Deep brain stimulation of the subthalamic nucleus versus levodopa challenge in Parkinson’s disease: measuring the on- and off-conditions with FDG-PET. J Neural Transm 109: 1257–1264. 12373559 10.1007/s00702-002-0696-5

[7] Hershey T, Black KJ, Carl JL, McGee-Minnich L, Snyder AZ, et al (2003) Long term treatment and disease severity change brain responses to levodopa in Parkinson’s disease. J Neurol Neurosurg Psychiatry 74: 844–851. 12810765 10.1136/jnnp.74.7.844 PMC1738560

[8] Jahanshahi M, Jenkins IH, Brown RG, Marsden CD, Passingham RE, et al (1995) Self-initiated versus externally triggered movements. I. An investigation using measurement of regional cerebral blood flow with PET and movement-related potentials in normal and Parkinson’s disease subjects. Brain 118 (Pt 4): 913–933. 10.1093/brain/118.4.913 7655888

[9] Jenkins IH, Fernandez W, Playford ED, Lees AJ, Frackowiak RS, et al (1992) Impaired activation of the supplementary motor area in Parkinson’s disease is reversed when akinesia is treated with apomorphine. Ann Neurol 32: 749–757. 1471865 10.1002/ana.410320608

[10] Playford ED, Jenkins IH, Passingham RE, Nutt J, Frackowiak RS, et al (1992) Impaired mesial frontal and putamen activation in Parkinson’s disease: a positron emission tomography study. Ann Neurol 32: 151–161. 1510355 10.1002/ana.410320206

[11] Rascol O, Sabatini U, Chollet F, Celsis P, Montastruc JL, et al (1992) Supplementary and primary sensory motor area activity in Parkinson’s disease. Regional cerebral blood flow changes during finger movements and effects of apomorphine. Arch Neurol 49: 144–148. 1736846 10.1001/archneur.1992.00530260044017

[12] Rascol O, Sabatini U, Fabre N, Brefel C, Loubinoux I, et al (1997) The ipsilateral cerebellar hemisphere is overactive during hand movements in akinetic parkinsonian patients. Brain 120 (Pt 1): 103–110. 10.1093/brain/120.1.103 9055801

[13] Sabatini U, Boulanouar K, Fabre N, Martin F, Carel C, et al (2000) Cortical motor reorganization in akinetic patients with Parkinson’s disease: a functional MRI study. Brain 123 (Pt 2): 394–403. 10.1093/brain/123.2.394 10648446

[14] Samuel M, Ceballos-Baumann AO, Blin J, Uema T, Boecker H, et al (1997) Evidence for lateral premotor and parietal overactivity in Parkinson’s disease during sequential and bimanual movements. A PET study. Brain 120 (Pt 6): 963–976. 10.1093/brain/120.6.963 9217681

[15] Thobois S, Dominey P, Decety J, Pollak P, Gregoire MC, et al (2000) Overactivation of primary motor cortex is asymmetrical in hemiparkinsonian patients. Neuroreport 11: 785–789. 10757520 10.1097/00001756-200003200-00026

[16] Haslinger B, Erhard P, Kampfe N, Boecker H, Rummeny E, et al (2001) Event-related functional magnetic resonance imaging in Parkinson’s disease before and after levodopa. Brain 124: 558–570. 11222456 10.1093/brain/124.3.558

[17] Peters S, Suchan B, Rusin J, Daum I, Koster O, et al (2003) Apomorphine reduces BOLD signal in fMRI during voluntary movement in Parkinsonian patients. Neuroreport 14: 809–812. 12858037 10.1097/00001756-200305060-00006

[18] Buhmann C, Glauche V, Sturenburg HJ, Oechsner M, Weiller C, et al (2003) Pharmacologically modulated fMRI–cortical responsiveness to levodopa in drug-naive hemiparkinsonian patients. Brain 126: 451–461. 12538411 10.1093/brain/awg033

[19] Kraft E, Loichinger W, Diepers M, Lule D, Schwarz J, et al (2009) Levodopa-induced striatal activation in Parkinson’s disease: a functional MRI study. Parkinsonism Relat Disord 15: 558–563. 19467909 10.1016/j.parkreldis.2009.02.005

[20] Liotti M, Ramig LO, Vogel D, New P, Cook CI, et al (2003) Hypophonia in Parkinson’s disease: neural correlates of voice treatment revealed by PET. Neurology 60: 432–440. 12578924 10.1212/wnl.60.3.432

[21] Narayana S, Fox PT, Zhang W, Franklin C, Robin DA, et al (2010) Neural correlates of efficacy of voice therapy in Parkinson’s disease identified by performance-correlation analysis. Hum Brain Mapp 31: 222–236. 19639554 10.1002/hbm.20859 PMC2811230

[22] Narayana S, Jacks A, Robin DA, Poizner H, Zhang W, et al (2009) A noninvasive imaging approach to understanding speech changes following deep brain stimulation in Parkinson’s disease. Am J Speech Lang Pathol 18: 146–161. 19029533 10.1044/1058-0360(2008/08-0004) PMC2779712

[23] Pinto S, Thobois S, Costes N, Le Bars D, Benabid AL, et al (2004) Subthalamic nucleus stimulation and dysarthria in Parkinson’s disease: a PET study. Brain 127: 602–615. 14736753 10.1093/brain/awh074

[24] Pinto S, Mancini L, Jahanshahi M, Thornton JS, Tripoliti E, et al (2011) Functional magnetic resonance imaging exploration of combined hand and speech movements in Parkinson’s disease. Mov Disord 26: 2212–2219. 21714000 10.1002/mds.23799 PMC3184369

[25] Rektorova I, Barrett J, Mikl M, Rektor I, Paus T (2007) Functional abnormalities in the primary orofacial sensorimotor cortex during speech in Parkinson’s disease. Mov Disord 22: 2043–2051. 17683056 10.1002/mds.21548

[26] Rektorova I, Mikl M, Barrett J, Marecek R, Rektor I, et al (2012) Functional neuroanatomy of vocalization in patients with Parkinson’s disease. J Neurol Sci 313: 7–12. 22078745 10.1016/j.jns.2011.10.020

[27] Sachin S, Senthil Kumaran S, Singh S, Goyal V, Shukla G, et al (2008) Functional mapping in PD and PSP for sustained phonation and phoneme tasks. J Neurol Sci 273: 51–56. 18675995 10.1016/j.jns.2008.06.024

[28] Agid Y, Graybiel AM, Ruberg M, Hirsch E, Blin J, et al (1990) The efficacy of levodopa treatment declines in the course of Parkinson’s disease: do nondopaminergic lesions play a role? Adv Neurol 53: 83–100. 1978522

[29] Braak H, Braak E, Yilmazer D, Schultz C, de Vos RA, et al.. (1995) Nigral and extranigral pathology in Parkinson’s disease. J Neural Transm Suppl 46: 15–31. 8821039

[30] Halliday G, Lees A, Stern M (2011) Milestones in Parkinson’s disease–clinical and pathologic features. Mov Disord 26: 1015–1021. 21626546 10.1002/mds.23669

[31] Kosaka K, Tsuchiya K, Yoshimura M (1988) Lewy body disease with and without dementia: a clinicopathological study of 35 cases. Clin Neuropathol 7: 299–305. 3224472

[32] MacDonald V, Halliday GM (2002) Selective loss of pyramidal neurons in the pre-supplementary motor cortex in Parkinson’s disease. Mov Disord 17: 1166–1173. 12465053 10.1002/mds.10258

[33] Henderson JM, Carpenter K, Cartwright H, Halliday GM (2000) Degeneration of the centre median-parafascicular complex in Parkinson’s disease. Ann Neurol 47: 345–352. 10716254

[34] McRitchie DA, Cartwright HR, Halliday GM (1997) Specific A10 dopaminergic nuclei in the midbrain degenerate in Parkinson’s disease. Exp Neurol 144: 202–213. 9126172 10.1006/exnr.1997.6418

[35] Bunton K, Keintz CK (2008) The Use of a Dual-Task Paradigm for Assessing Speech Intelligibility in Clients with Parkinson Disease. J Med Speech Lang Pathol 16: 141–155. 21637738 PMC3104935

[36] Dromey C, Jarvis E, Sondrup S, Nissen S, Foreman KB, et al (2010) Bidirectional interference between speech and postural stability in individuals with Parkinson’s disease. Int J Speech Lang Pathol 12: 446–454. 20586526 10.3109/17549507.2010.485649

[37] Ho AK, Iansek R, Bradshaw JL (2002) The effect of a concurrent task on Parkinsonian speech. J Clin Exp Neuropsychol 24: 36–47. 11935422 10.1076/jcen.24.1.36.972

[38] LaPointe LL, Stierwalt JA, Maitland CG (2010) Talking while walking: Cognitive loading and injurious falls in Parkinson’s disease. Int J Speech Lang Pathol 12: 455–459. 20632845 10.3109/17549507.2010.486446

[39] Benecke R, Rothwell JC, Day BL, Dick JP, Marsden CD (1986) Motor strategies involved in the performance of sequential movements. Exp Brain Res 63: 585–595. 3758270 10.1007/BF00237481

[40] Benecke R, Rothwell JC, Dick JP, Day BL, Marsden CD (1986) Performance of simultaneous movements in patients with Parkinson’s disease. Brain 109 (Pt 4): 739–757. 10.1093/brain/109.4.739 3730813

[41] Brown RG, Jahanshahi M (1998) An unusual enhancement of motor performance during bimanual movement in Parkinson’s disease. J Neurol Neurosurg Psychiatry 64: 813–816. 9647320 10.1136/jnnp.64.6.813 PMC2170109

[42] Brown RG, Jahanshahi M, Marsden CD (1993) The execution of bimanual movements in patients with Parkinson’s, Huntington’s and cerebellar disease. J Neurol Neurosurg Psychiatry 56: 295–297. 8459247 10.1136/jnnp.56.3.295 PMC1014865

[43] Horstink MW, Berger HJ, van Spaendonck KP, van den Bercken JH, Cools AR (1990) Bimanual simultaneous motor performance and impaired ability to shift attention in Parkinson’s disease. J Neurol Neurosurg Psychiatry 53: 685–690. 2213046 10.1136/jnnp.53.8.685 PMC488173

[44] Palmer SJ, Eigenraam L, Hoque T, McCaig RG, Troiano A, et al (2009) Levodopa-sensitive, dynamic changes in effective connectivity during simultaneous movements in Parkinson’s disease. Neuroscience 158: 693–704. 18722512 10.1016/j.neuroscience.2008.06.053

[45] Wu T, Wang L, Hallett M, Chen Y, Li K, et al (2011) Effective connectivity of brain networks during self-initiated movement in Parkinson’s disease. Neuroimage 55: 204–215. 21126588 10.1016/j.neuroimage.2010.11.074

[46] Wu T, Hallett M (2008) Neural correlates of dual task performance in patients with Parkinson’s disease. J Neurol Neurosurg Psychiatry 79: 760–766. 18006652 10.1136/jnnp.2007.126599

[47] Gibb WR, Lees AJ (1988) The relevance of the Lewy body to the pathogenesis of idiopathic Parkinson’s disease. J Neurol Neurosurg Psychiatry 51: 745–752. 2841426 10.1136/jnnp.51.6.745 PMC1033142

[48] Fahn S, Elton RL, motUd c (1987) Unified Parkinson’s Disease Rating Scale. In: Fahn S, Marsden, C.D, Calne, D.B., editor. Recent developments in Parkinson’s disease. Florham Park: MacMillan. 153–164.

[49] Friston KJ, Holmes AP, Worsley KJ, Poline JP, Frith CD, et al (1994) Statistical parametric maps in functional imaging: A general linear approach. Hum Brain Mapp 2: 189–210.

[50] Talairach J, Tournoux P (1988) Co-planar stereotaxic atlas of the human brain. New-York, NY: Thième Medical Publishers.

[51] Ballanger B, Baraduc P, Broussolle E, Le Bars D, Desmurget M, et al (2008) Motor urgency is mediated by the contralateral cerebellum in Parkinson’s disease. J Neurol Neurosurg Psychiatry 79: 1110–1116. 18356249 10.1136/jnnp.2007.141689

[52] Thobois S, Ballanger B, Baraduc P, Le Bars D, Lavenne F, et al (2007) Functional anatomy of motor urgency. Neuroimage 37: 243–252. 17553705 10.1016/j.neuroimage.2007.04.049

[53] Wu T, Hallett M (2005) A functional MRI study of automatic movements in patients with Parkinson’s disease. Brain 128: 2250–2259. 15958505 10.1093/brain/awh569

[54] Yu H, Sternad D, Corcos DM, Vaillancourt DE (2007) Role of hyperactive cerebellum and motor cortex in Parkinson’s disease. Neuroimage 35: 222–233. 17223579 10.1016/j.neuroimage.2006.11.047 PMC1853309

[55] Asanuma K, Tang C, Ma Y, Dhawan V, Mattis P, et al (2006) Network modulation in the treatment of Parkinson’s disease. Brain 129: 2667–2678. 16844713 10.1093/brain/awl162 PMC4459513

[56] Trost M, Su S, Su P, Yen RF, Tseng HM, et al (2006) Network modulation by the subthalamic nucleus in the treatment of Parkinson’s disease. Neuroimage 31: 301–307. 16466936 10.1016/j.neuroimage.2005.12.024 PMC4454374

[57] Turner RS, Grafton ST, McIntosh AR, DeLong MR, Hoffman JM (2003) The functional anatomy of parkinsonian bradykinesia. Neuroimage 19: 163–179. 12781736 10.1016/s1053-8119(03)00059-4

[58] Payoux P, Remy P, Damier P, Miloudi M, Loubinoux I, et al (2004) Subthalamic nucleus stimulation reduces abnormal motor cortical overactivity in Parkinson disease. Arch Neurol 61: 1307–1313. 15313852 10.1001/archneur.61.8.1307

[59] Thobois S, Dominey P, Fraix V, Mertens P, Guenot M, et al (2002) Effects of subthalamic nucleus stimulation on actual and imagined movement in Parkinson’s disease : a PET study. J Neurol 249: 1689–1698. 12529791 10.1007/s00415-002-0906-y

[60] Flasskamp A, Kotz SA, Schlegel U, Skodda S (2012) Acceleration of syllable repetition in Parkinson’s disease is more prominent in the left-side dominant patients. Parkinsonism Relat Disord 18: 343–347. 22169630 10.1016/j.parkreldis.2011.11.021

[61] Ackermann H (2008) Cerebellar contributions to speech production and speech perception: psycholinguistic and neurobiological perspectives. Trends Neurosci 31: 265–272. 18471906 10.1016/j.tins.2008.02.011

[62] Astrom M, Tripoliti E, Hariz MI, Zrinzo LU, Martinez-Torres I, et al (2010) Patient-specific model-based investigation of speech intelligibility and movement during deep brain stimulation. Stereotact Funct Neurosurg 88: 224–233. 20460952 10.1159/000314357 PMC3214825

[63] Tripoliti E, Zrinzo L, Martinez-Torres I, Tisch S, Frost E, et al (2008) Effects of contact location and voltage amplitude on speech and movement in bilateral subthalamic nucleus deep brain stimulation. Mov Disord 23: 2377–2383. 18785648 10.1002/mds.22296

[64] De Letter M, Santens P, De Bodt M, Van Maele G, Van Borsel J, et al (2007) The effect of levodopa on respiration and word intelligibility in people with advanced Parkinson’s disease. Clin Neurol Neurosurg 109: 495–500. 17509751 10.1016/j.clineuro.2007.04.003

[65] De Letter M, Santens P, Estercam I, Van Maele G, De Bodt M, et al (2007) Levodopa-induced modifications of prosody and comprehensibility in advanced Parkinson’s disease as perceived by professional listeners. Clin Linguist Phon 21: 783–791. 17882694 10.1080/02699200701538181

[66] Skodda S, Visser W, Schlegel U (2010) Short- and long-term dopaminergic effects on dysarthria in early Parkinson’s disease. J Neural Transm 117: 197–205. 20012657 10.1007/s00702-009-0351-5

[67] Albin RL, Young AB, Penney JB (1989) The functional anatomy of basal ganglia disorders. Trends Neurosci 12: 366–375. 2479133 10.1016/0166-2236(89)90074-x

[68] Alexander GE, Crutcher MD (1990) Functional architecture of basal ganglia circuits: neural substrates of parallel processing. Trends Neurosci 13: 266–271. 1695401 10.1016/0166-2236(90)90107-l

[69] Alexander GE, Crutcher MD, DeLong MR (1990) Basal ganglia-thalamocortical circuits: parallel substrates for motor, oculomotor, “prefrontal" and “limbic" functions. Prog Brain Res 85: 119–146. 2094891

[70] Kopell BH, Rezai AR, Chang JW, Vitek JL (2006) Anatomy and physiology of the basal ganglia: implications for deep brain stimulation for Parkinson’s disease. Mov Disord 21 Suppl 14 S238–246. 16810674 10.1002/mds.20958

[71] Obeso JA, Rodriguez-Oroz MC, Rodriguez M, Lanciego JL, Artieda J, et al (2000) Pathophysiology of the basal ganglia in Parkinson’s disease. Trends Neurosci 23: S8–19. 11052215 10.1016/s1471-1931(00)00028-8

[72] Joel D, Weiner I (1994) The organization of the basal ganglia-thalamocortical circuits: open interconnected rather than closed segregated. Neuroscience 63: 363–379. 7891852 10.1016/0306-4522(94)90536-3

[73] Klawans HL (1986) Individual manifestations of Parkinson’s disease after ten or more years of levodopa. Mov Disord 1: 187–192. 3504244 10.1002/mds.870010304

[74] Pinto S, Ozsancak C, Tripoliti E, Thobois S, Limousin-Dowsey P, et al (2004) Treatments for dysarthria in Parkinson’s disease. Lancet Neurol 3: 547–556. 15324723 10.1016/S1474-4422(04)00854-3

[75] Clarici A, Fabbro F, Bava A, Daro V (1994) Effects of speaking speed on cerebral lateralization of speech assessed by a dual-task interference paradigm. Percept Mot Skills 78: 947–953. 8084716 10.1177/003151259407800349

[76] McFarland K, Ashton R, Rich A, Donald AM (1989) Lateralised dual-task performance: the effect of muscular-repositioning. Cortex 25: 433–447. 2805729 10.1016/s0010-9452(89)80057-7

[77] Simon TJ, Sussman HM (1987) The dual task paradigm: speech dominance or manual dominance? Neuropsychologia 25: 559–569. 3683813 10.1016/0028-3932(87)90080-7

[78] Rochester L, Nieuwboer A, Baker K, Hetherington V, Willems AM, et al (2008) Walking speed during single and dual tasks in Parkinson’s disease: which characteristics are important? Mov Disord 23: 2312–2318. 18816800 10.1002/mds.22219

[79] Dagher A, Owen AM, Boecker H, Brooks DJ (2001) The role of the striatum and hippocampus in planning: a PET activation study in Parkinson’s disease. Brain 124: 1020–1032. 11335704 10.1093/brain/124.5.1020

[80] Rowe J, Stephan KE, Friston K, Frackowiak R, Lees A, et al (2002) Attention to action in Parkinson’s disease: impaired effective connectivity among frontal cortical regions. Brain 125: 276–289. 11844728 10.1093/brain/awf036

[81] Brown P (2006) Bad oscillations in Parkinson’s disease. J Neural Transm Suppl: 27–30. 10.1007/978-3-211-45295-0_6 17017505

